# Naltrexone alters responses to social and physical warmth: implications for social bonding

**DOI:** 10.1093/scan/nsz026

**Published:** 2019-04-12

**Authors:** Tristen K Inagaki, Laura I Hazlett, Carmen Andreescu

**Affiliations:** 1 Department of Psychology, University of Pittsburgh, Pittsburgh, PA, USA; 2 Department of Psychology, University of California, Los Angeles, CA, USA; 3 Department of Psychiatry, University of Pittsburgh School of Medicine, Pittsburgh, PA, USA

**Keywords:** brain opioid theory, social reward, human, social warmth, social attachments

## Abstract

Socially warm experiences, when one feels connected to others, have been linked with physical warmth. Opioids, hypothesized to support social bonding with close others and, separately, physical warmth, may underlie both experiences. In order to test this hypothesis, 80 participants were randomly assigned to the opioid antagonist, naltrexone or placebo before neural and emotional responses to social and physical warmth were collected. Social and physical warmth led to similar increases in ventral striatum (VS) and middle-insula (MI) activity. Further, feelings of social connection were positively related to neural activity to social warmth. However, naltrexone (*vs* placebo) disrupted these effects by (i) reducing VS and MI activity to social and physical warmth, (ii) erasing the subjective experience–brain association to social warmth and (iii) disrupting the neural overlap between social and physical warmth. Results provide additional support for the theory that social and physical warmth share neurobiological, opioid receptor-dependent mechanisms and suggest multiple routes by which social connections may be maintained.

## Introduction

Close social connections and the feelings that come from being connected to other individuals are of utmost importance. Decades of research confirms that humans need close social connections to grow and thrive (Bowlby, 1988; Leary and Baumeister, 1995; Holt-Lunstad *et al.,* 2010). However, relative to its suggested importance, feelings of social connection with our closest loved ones and the neurochemical, neural and psychological mechanisms that support such feelings are poorly understood. Therefore, we examine in the current study the causal role of opioids in feelings of social connection, neural activity to an experience of social connection and the potential overlapping contribution of physical warmth to social connection.

## Opioids and social connection

Due to the importance of social connection for well-being, basic homeostatic mechanisms that maintain individuals at an optimal level of functioning may be involved in the maintenance of social bonds (Panksepp *et al.,* 1980a; Panksepp, 1998). One candidate system that has received increasing attention in recent years is the endogenous opioid system, best known for its role in pain relief and pleasure (Leknes and Tracey, 2008). According to the brain opioid theory of social attachment, opioids contribute to emotional responding within close relationships and to the behavior or feelings that might promote further bonding (Panksepp *et al.,* 1980a; Machin and Dunbar, 2011; Inagaki, 2018). Evidence for the theory comes primarily from animal research, which has shown that pharmacologic manipulations of the opioid system alter social-bonding behavior [reviewed in Machin and Dunbar (2011) and Loseth *et al.* (2014)]. For instance, blocking endogenous opioid activity (*vs* placebo; Panksepp *et al.*, 1980b; Shayit *et al.*, 2003) or using genetic knockout models (Moles *et al.,* 2004) reduced social-bonding behavior. Conversely, naltrexone (*vs* placebo) has also been shown to increase social-bonding behavior (e.g. Martel *et al.*, 1993; Schino and Troisi, 1992). Thus, animal research shows that opioids affect social behavior, suggesting opioids may also have implications for the affective experience of social connection in humans.

The relatively smaller human literature on opioids and social bonding also suggests opioids alter the affective experience related to social bonding (Inagaki, 2018). Naltrexone (*vs* placebo) reduced affiliative feelings to lab tasks designed to promote feelings of social connection to close others (Inagaki *et al.,* 2015*,*2016a) and more general affiliative stimuli of strangers (Depue and Morrone-Strupinsky, 2005; Schweiger *et al.,* 2013; Chelnokova *et al.,* 2014). Outside of the lab, daily feelings of social connection are also reduced by naltrexone (*vs* placebo; Inagaki *et al.*, 2016a).

Opioids are theorized to contribute to social bonding via the system’s actions on distinct brain regions. Indeed, the first indirect evidence for opioids and social bonding came from observations that the neural regions most densely concentrated in opioid receptors (Cross *et al.,* 1987; Zubieta *et al.,* 2001) are also the regions that contribute to social bonding. These regions include the ventral striatum (VS), middle insula (MI), ventral tegmental area (VTA), anterior cingulate cortex (ACC) and orbitofrontal cortex (OFC; Bartels and Zeki, 2004; Aron *et al.,* 2005; Acevedo *et al.,* 2012; Inagaki and Eisenberger, 2013; Atzil *et al.,* 2017). Positron emission tomography (PET) imaging, which examines opioid activity *in vivo*, confirmed initial observational evidence. Endogenous opioid release in males (i.e. decreased binding of the μ-opioid-specific ligand [^11^C]carfentanil) increased in the insula and cingulate cortices after a laughter manipulation with friends (Manninen *et al.,* 2017). In addition, greater laughter between friends was associated with opioid-receptor binding in the VS, OFC, anterior and middle cingulate cortices at baseline. Similarly, increased desire for social interaction was associated with left VS binding to learning that one is liked by an opposite-sex stranger (Hsu *et al.,* 2013). Collectively, results from animals and humans point to the opioid system as a promising contributor to social bonding.

## Social–physical warmth

Connecting with others is often described as a heart-warming or warm experience. More than metaphor, physical warmth, and the thermoregulatory pathways that keep individuals at an optimally warm core body temperature, may also contribute to experiences of social warmth, the experience of feeling loved by, cared for and connected to other people (Panksepp, 1998; Inagaki *et al.,* 2016b). In particular, we have proposed that humans have an innate system that both supports social warmth and concurrently maintains core temperature for survival purposes (Inagaki *et al.,* 2016b). The association between social and physical warmth may have then been strengthened by our very first social encounter: that between infant and caregiver. Warmth may have simultaneously signaled the proximity of a caregiver (physical warmth) and of potential care or connection (social warmth) such that later in life there may be a bidirectional relationship where activating either physical or social warmth might similarly activate the other.

In support of this hypothesis, cutaneous warmth has been shown to affect social warmth and the reverse (though results are not uniform; Lynott *et al.*, 2014). For example, holding a warm object (*vs* room temperature object) led to increases in affiliative feelings (IJzerman and Semin, 2009; Fay and Maner, 2012). In the other direction, experimentally increasing feelings of social connection led to increases in feelings of warmth (Inagaki and Eisenberger, 2013), whereas experimentally decreasing feelings of social connection led to decreases in perceptions of warmth (Zhong and Leonardelli, 2008).

More direct evidence for shared mechanisms comes from neuroimaging work in humans. Neural activity to a socially warm experience shared overlapping neural activity with physical warmth but not another pleasant task (Inagaki and Eisenberger, 2013). Specifically, activity in the VS and MI, regions known to activate to warm thermal stimuli (e.g. Rolls *et al.*, 2008) and, separately, to close social connections (Bartels and Zeki, 2004; Aron *et al.,* 2005; Acevedo *et al.,* 2012; Inagaki and Eisenberger, 2013; Atzil *et al.,* 2017), showed similar responses to both kinds of experiences. Thus, it is possible that these regions contribute to the convergence of the subjective experiences of warmth and social connection.

## Opioids and physical warmth

Opioids may also contribute to physical warmth, although current literature to support this hypothesis is sparse. In animals, opioids mediate changes in core body temperature (Clark, 1979; Adler *et al.,* 1988) with opioid agonists injected into the lateral cerebral ventricle leading to an increase in core body temperature (Clark *et al.,* 1983) and opioid antagonists decreasing body temperature (Handler *et al.,* 1992). Similarly, in humans, exogenously administered opiates alter correlates of thermoregulatory function (Martin *et al.,* 1973; Kurz *et al.,* 1995). As reviewed above, physically warm experiences led to increases in feelings of social connection. Therefore, we expect opioids to alter the effect of physical warmth on feelings of social connection. That is, opioids might further affect social connection by increasing felt social connection when experiencing physical warmth.

In the only study to examine the contribution of opioids to physical-warmth-induced feelings of social connection, participants took naltrexone and placebo before evaluating warm and cool stimuli (Inagaki *et al.,* 2015). As expected, holding a warm (*vs* cool) object increased feelings of social connection. Naltrexone, however, specifically reduced feelings of social connection to holding the warm but not the cool object. Thus, it is plausible that opioids similarly contribute to social and physical warmth. However, whether opioids affect neural activity to both social and physical warmth and the potential link between the two experiences is currently unknown.

## The current study

One way to examine the causal contribution of opioids to social bonding is to pharmacologically manipulate opioids. Therefore, the current study used a double-blind, randomized, placebo-controlled design with the opioid antagonist, oral naltrexone, to test the following primary aims: (i) the effect of naltrexone on feelings of social connection and VS and MI activity to a socially warm experience; (ii) the effect of naltrexone on the same outcomes to a physically warm experience; and (iii) the effect of naltrexone on the neural overlap between social and physical warmth. Based on the existing animal and human research on the contribution of opioids to both experiences of social connection and physical warmth, naltrexone (*vs* placebo) was hypothesized to reduce feelings of social connection and VS and MI activity to social warmth and physical warmth. Further, naltrexone was hypothesized to disrupt the neural overlap between social and physical warmth.

Inherent in the perspective outlined above is that opioids and physical warmth are particularly relevant for social bonding that occurs with close others. That is, opioids likely contribute to the experience of connecting with friends, family and other people one feels particularly close to. Similarly, warmth may signal the proximity of close others, such as a responsive caregiver or loved one, whereas cold may signal the absence of close others. Therefore, a final aim of the current study was to assess naltrexone’s effect on responses to both close others and strangers and, separately, warm and cool stimuli. Naltrexone was not expected to affect responses to strangers or cool stimuli.

## Materials and method

### Participants

Recruitment took place via flyers and a posting to a research registry. A total of 82 participants were deemed eligible and were then randomized to naltrexone or placebo by an individual unassociated with the study. Two participants did not complete the study (see the CONSORT flow diagram as Supplementary Figure 1), leaving a final sample of 80 participants (*M age* = 22.39, s.d. = 3.362) who were randomly assigned to naltrexone (*n* = 40, 21 females) or placebo (*n* = 40, 27 females). For information about screening, see Supplementary Materials. The University of Pittsburgh’s Investigational Drug Service compounded study drugs and the PI’s (T.K.I.) lab dispensed drugs. Participants were compensated $90 for completing the study.

The study was registered on the U.S. National Institutes of Health Clinical Trials registry as NCT02818036. Procedures were performed between September 2016 and June 2018 following approval from the University of Pittsburgh’s Human Research Protection Office (including written consent).

### Overview of experimental session

Details regarding the study procedures are reported elsewhere (Inagaki *et al.,* unpublished data) and in the Supplementary Materials, but none of the data or tasks reported here have previously been reported. During the experimental session, participants were randomly assigned to 50 mg of oral naltrexone or placebo. Approximately 60 min following drug administration, when naltrexone shows peak effects (Lee *et al.,* 1988), participants completed three tasks in the fMRI scanner in the following order: social warmth task, picture task (see Supplementary Materials) and physical warmth task. Finally, post-scan self-reports were collected. Physical symptoms and the distress from any symptoms are reported under Supplementary Materials.

### Neuroimaging measures

#### Social warmth task

For the social warmth task, experimenters collected messages from each of our participants’ close others in order to create personalized tasks. For pre-scan message collection procedures, see Supplementary Materials. Participants were told that the experimenters had asked their friends and family members to provide 12 messages each and that they would be reading the messages in the scanner (close other condition). In addition, participants read randomly selected messages from a previous participant’s friends and family members (stranger condition). No specific instructions were given as to what to think about while reading. Blocks began with a 2-s cue explaining whom the messages were from [e.g. The following messages are from Ed (example close other cue); the following messages are from a previous participant (stranger cue)] followed by three messages, either from a single close other or a stranger, presented for 5 s each. Blocks were separated by 6 s of fixation crosshair, and close other and stranger blocks were randomized. Presented were 40 sets of messages (20 close other; 20 stranger) over 2 functional runs. Examples of messages from close others in the current study include the following: *‘*I really appreciate the way you love and support me; our connection is one of the most beautiful things in my life.*’*; *‘*You are endlessly funny and bring joy to everyone around you.’; *‘*I love that I can always call on you to experience new things with.*’*; *‘*You light up my entire world.*’*

#### Physical warmth task

For the physical warmth task, participants held warm and cool therapeutic packs and a neutral, room temperature object (ball) ostensibly for a product evaluation task (Kang *et al.,* 2010). Blocks began with a 3-s cue indicating which object would be presented. The experimenter then placed one of the three objects in the participant’s left hand where they held the item for 10.5 s. Each block was separated by 7 s during which time no stimulation was delivered. Each of the three objects was presented 10 times over 2 functional runs, no object was presented twice in a row and the order of presentation was randomized for each participant.

### Post-scan self-report measures

#### Social warmth task

After exiting the scanner, participants re-read the messages they had read in the scanner and rated their feelings of social connection on a 1 (not at all) to 7 (very) scale using the same measure used in our previous study on the effect of naltrexone on feelings of social connection to the same task (Inagaki *et al.*, 2015; i.e. ‘When you were reading the last set of messages: how connected/touched/warm did you feel?’). Ratings to the three items were combined to create our measure of feelings of connection (α for close others = 0.823; α for strangers = 0.874). Ratings for two participants were not included in final analyses (Supplementary Figure 1).

#### Physical warmth task

For the physical warmth task, participants rated the thermal intensity (how warm/cool did the item feel?) and their feelings of social connection to the three objects on a 1 (not at all) to 7 (very) scale (When you were holding the warm pack/cool pack/ball, how connected/touched/warm did you feel when holding this item?; *α* for combined three-item measure = 0.693–0.881). In addition, to enhance the cover story that the aim of the task was to evaluate commercial products, participants were asked how likely they would be to recommend the product to a friend. Ratings to the physical warmth task were missing from one participant in the naltrexone condition.

#### fMRI data acquisition

Participants were scanned at the MR Research Center at the University of Pittsburgh on a Siemens 3 T MAGNETOM Prisma MRI Scanner. A magnetization-prepared rapid gradient echo scan (MPRAGE; TR/TE = 5000/2.97 ms, flip angle = 4°, 256 $\times$ 256 matrix, 177 sagittal slices, FOV = 258; 1 mm thick) was acquired prior to functional scans to aid in data registration. Participants then completed two runs of the social warmth task (7 min, 53 s each), one run of the picture task (reported separately) and two runs of the physical warmth task (5 min, 25 s each), (T_2_*-weighted gradient-echo covering 60 axial slices, TR/TE = 1000/28 ms; flip angle = 55°; 112 $\times$ 112 matrix; FOV = 220 mm; 2 mm thick).

### Statistical analyses

#### Neuroimaging data

Imaging data were pre-processed in SPM8 (Wellcome Department of Imaging Neuroscience, London) using the DARTEL procedure. After motion correction, images were realigned to the MPRAGE, warped into Montreal Neurological Institute (MNI) space and smoothed with a 5-mm Gaussian kernel full-width, half-maximum. Contrasts for the main comparisons of interest were computed (social warmth task: messages from close others > strangers; physical warmth task: warm > cool; warm > neutral; cool > neutral) at the first level and then brought to the group level of analysis. Three participants were removed from imaging analyses (Supplementary Figure 1), leaving a final imaging sample of 77 (placebo *n* = 37, naltrexone *n* = 40).

Group level results were examined in two ways. To confirm the regions-of-interest (ROI) analyses described below and to replicate our previous findings using similar social and physical warmth tasks (Inagaki and Eisenberger, 2013), whole-brain analyses were run. Whole-brain results are reported under Supplementary Materials.

The main aim of the current study is to assess the effect of naltrexone on neural activity to both social and physical warmth. As such, an a priori, independently defined structural mask of the only two regions to show similar activity to social and physical warmth in our previous work (Inagaki and Eisenberger, 2013) was created. Specifically, anatomical masks of the VS (Inagaki and Eisenberger, 2012; Inagaki *et al.,* 2016c) and MI were created with the MarsBar Toolbox (Brett *et al.,* 2002). Due to directional hypotheses and convention, ROI analyses are thresholded at *P* < .05, one-tailed.

#### Post-scan self-report data

Self-report data were analyzed in SPSS v.25. To assess changes in feelings of social connection to the social and physical warmth tasks as a function of naltrexone, self-reports were submitted to repeated measures analysis of variance with drug (naltrexone *vs* placebo) as a between-subjects factor and social target (messages from close others *vs* strangers) or temperature (warm *vs* cool) as within-subjects factors. For ease of interpretation and to remain consistent with the way the physical warmth task has been analyzed and reported on previously (Inagaki *et al.,* 2015), difference scores were calculated, subtracting mean ratings to the neutral object from mean ratings to each of the other temperature conditions (warm and cool). Significant interactions were further evaluated with independent samples *t*-tests to assess differences between drug conditions.

#### Associations between feelings of social connection and neural activity

Correlational analyses assessed associations between feelings of social connection and VS and MI activity to the social and physical warmth tasks. 95% confidence intervals (CI) were estimated using the bias corrected and accelerated percentile bootstrap method with 1000 random samples with replacement.

#### Shared neural activity: social and physical warmth

Previous research suggests that social and physical warmth share neurobiological mechanisms (Inagaki and Eisenberger, 2013; Inagaki *et al.,* 2015). To examine this possibility, we ran conjunction analyses on the whole brain in the placebo group and the naltrexone group separately. Specifically, we tested the primary contrasts from social warmth (messages from close others > strangers) and physical warmth (warm > neutral) against the conjunction null that identifies neural regions active during both tasks (Nichols *et al.,* 2005). We expected to see similarities in the VS and MI to the two tasks during placebo but not during naltrexone. Conjunction analyses were thresholded at a false discovery rate (FDR) of <0.05 with a cluster size threshold of 16 voxels.

## Results

### Effect of naltrexone on neural activity to social warmth

To examine the effect of naltrexone on neural activity to the social warmth task, analyses were constrained to the a priori defined VS and MI mask. VS and MI activity was greater to messages from close others than strangers (*left t*(76) = 6.138, *P* < 0.001; *right t*(76) = 4.493, *P* < 0.001). As confirmatory evidence of the results corrected at ROI, messages from close others (*vs* strangers) also lead to greater VS and MI activity corrected at whole brain (Supplementary Materials).

**Fig. 1:**
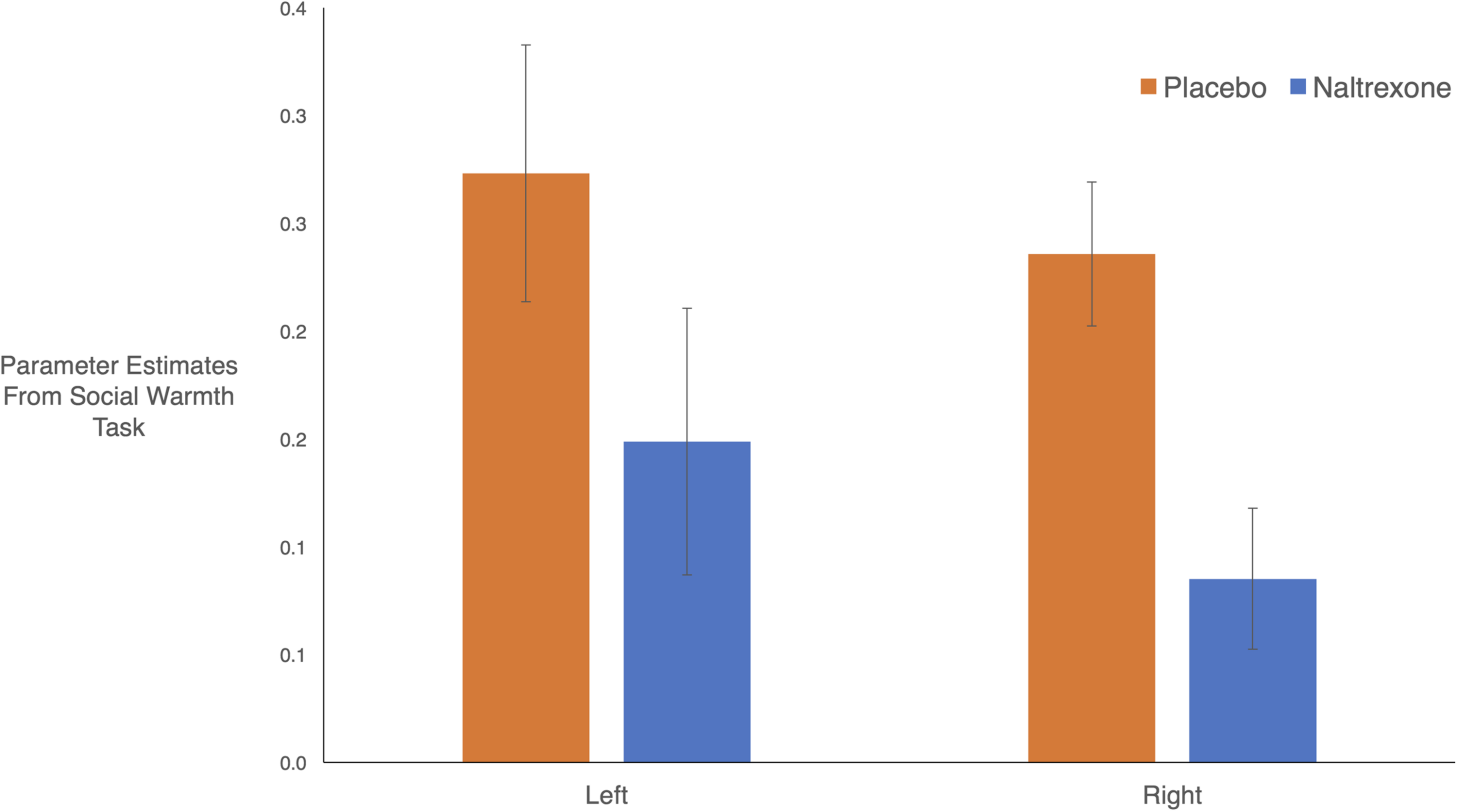
Neural activity in response to social warmth task. Naltrexone (*vs* placebo) reduced VS and MI activity to messages from close others (*vs* strangers). Mean parameter estimates from left- and right-sided activity pictured above.

Naltrexone, however, reduced VS and MI activity to the social warmth task as compared to placebo (*left t*(75) = 1.859, *P* = 0.034; *right t*(75) = 2.198, *P* = 0.016; Figure 1). In other words, naltrexone reduced VS and MI activity to messages from one’s own friends, family and romantic partners, consistent with the current hypotheses.

### Effect of naltrexone on feelings of social connection to social warmth

As a manipulation check, feelings of social connection to reading the messages were first assessed ignoring drug condition. Feelings of social connection were indeed greater to messages from close others (*M* = 4.671, s.d. = 0.871) than strangers [*M* = 1.966, s.d. = 0.720; *F*(1,76) = 612.614, *P* < 0.001], mirroring the pattern from the neural data and confirming the task elicited the intended feelings. The effect of drug on feelings of social connection to the messages was in the expected direction but did not reach statistical significance [*F*(1, 76) = 2.017, *P* = 0.080]. For the descriptive statistics, see Supplementary Table 2.

### Associations between feelings of social connection and neural activity to social warmth

**Fig. 2:**
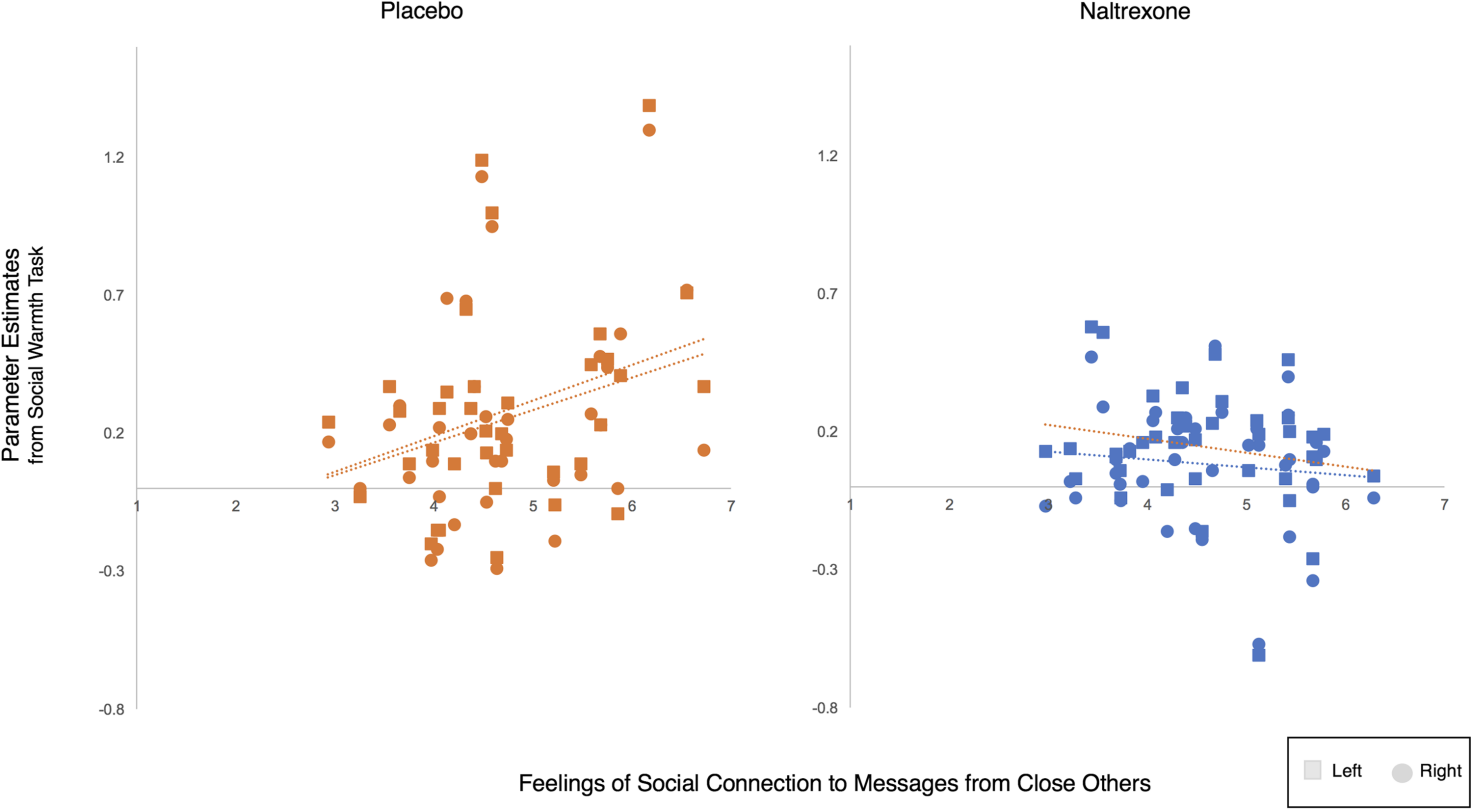
Associations between feelings of social connection and neural activity in response to the social warmth task. There was a positive correlation between feelings of social connection and activity in the VS and MI to the social warmth task such that greater feelings of social connection to close others were associated with greater VS and MI activity to messages from close others (*vs* strangers). The same association was absent in the naltrexone group, suggesting naltrexone erased the subjective experience–brain relationship.

In the placebo group, feelings of social connection were positively related to neural activity to the social warmth task (left: *r* = 0.325, *P* = 0.027, 95% CI = [0.027, 0.577]; right: *r* = 0.290, *P* = 0.043, 95% CI = [0.006, 0.541]; Figure 2). Thus, greater feelings of social connection were associated with greater VS and MI activity to reading messages from close others (*vs* strangers). However, there was no association between feelings of social connection and neural activity to the social warmth task in the naltrexone group (left: *r* = −0.200, *P* = 0.112, 95% CI = [−0.426, 0.091]; right: *r* = −0.114, *P* = 0.245, 95% CI = [−0.370, 0.183]). Further, the correlations in the placebo group were different from the correlations in the naltrexone group (left: *z* = 2.24, *P* = 0.013; right: *z* = 1.71, *P* = 0.043), suggesting naltrexone erased the subjective experience–brain relationship that was present in the placebo group.

### Effect of naltrexone on neural activity to physical warmth

**Fig. 3:**
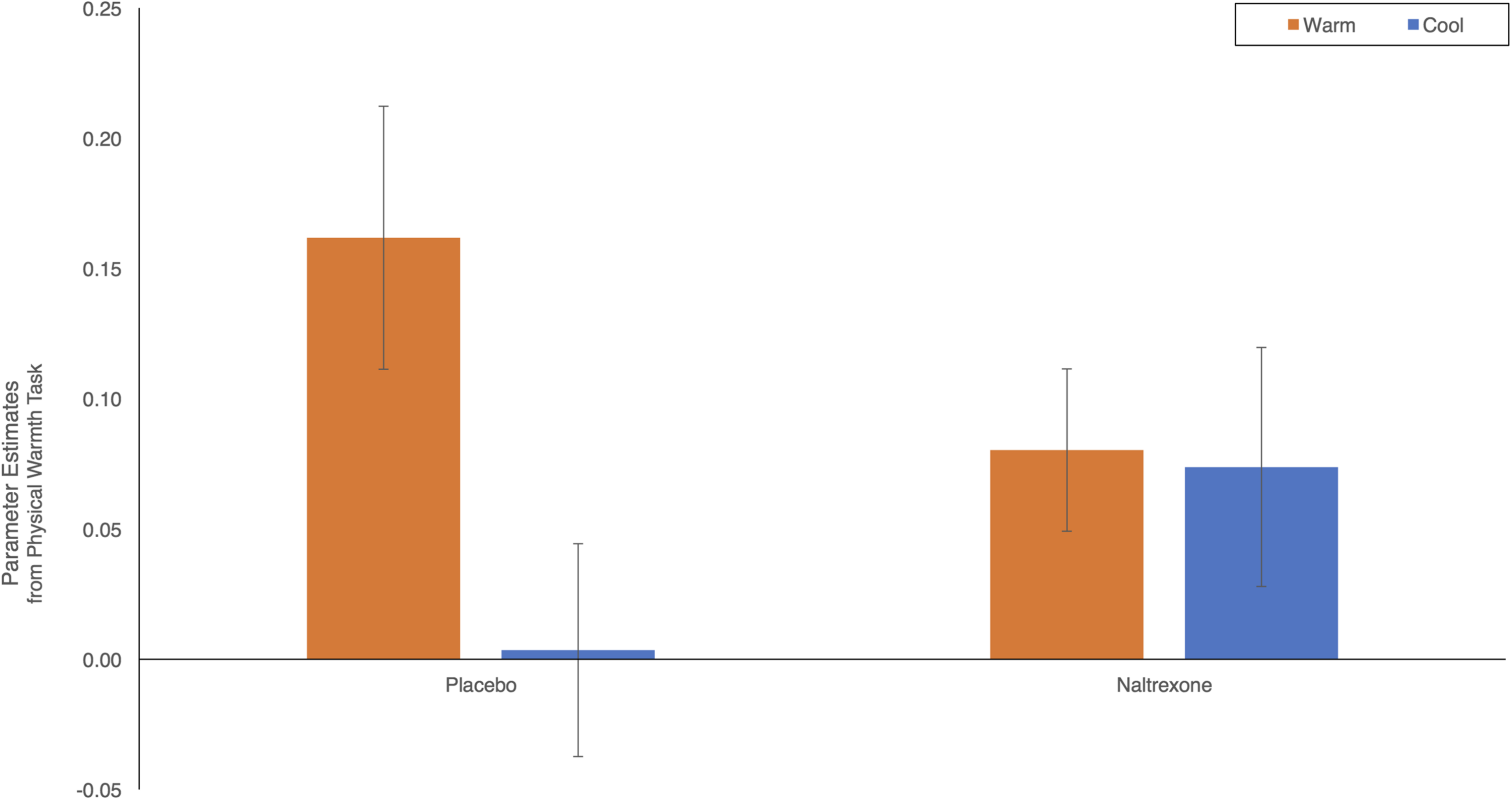
Neural activity in response to the physical warmth task. Examining neural responding to the physical warmth task revealed an interaction between drug and temperature. VS and MI activity was greater to holding a warm object compared to holding a cool object in the placebo group, but there was no difference in the naltrexone group. Parameter estimates from left-sided VS and MI activity plotted on the y-axis.

The effect of naltrexone on VS and MI activity to physical warmth revealed a pattern similar to the social warmth task. Once again mirroring results corrected at the whole brain (Supplementary Materials), there was greater VS and MI activity to the warm than the neutral and cool objects. Importantly, the main effect of temperature was qualified by an interaction [left: *F*(1, 75) = 5.027, *P* = 0.014; right: *F*(1, 75) = 2.624, *P* = 0.055; Figure 3] such that the difference between VS and MI activity to the warm and cool objects was eliminated by naltrexone. Thus, VS and MI activity was greater to the warm than cool object in the placebo group [left: *t*(36) = 2.672, *P* = .006; right: *t*(36) = 1.991, *P* = .027], but in the naltrexone group, there was no difference in neural responding [left: *t*(39) = .182, *P* = .429; right: *t*(39) = .165, *P* = .435]. In other words, naltrexone reduced VS and MI activity to physical warmth. Interactions between drug and temperature for the comparison of the warm to neutral object were not significant for neural results corrected at the ROI threshold (*Ps* > 0.06).

### Effect of naltrexone on feelings of social connection to physical warmth

Naltrexone’s effect on self-reported feelings was evaluated with a drug (naltrexone *vs* placebo) $\times$ temperature (warm *vs* cool) interaction. In a replication of prior work (Inagaki and Eisenberger, 2013; Inagaki *et al.,* 2015), a main effect of temperature [*F*(1,77) = 146.745, *P* < .001] revealed that the warm object led to greater feelings of social connection (*M* = 1.612, s.d. = 1.120) than the cool object [*M* = .135, s.d. = 0.979, *t*(78) = 12.004, *P* < 0.001]. Importantly, the main effect was qualified by a marginal interaction with drug [*F*(1, 77) = 2.721, *P* = 0.052]. Given the primary hypothesis that naltrexone might affect physical warmth-induced feelings of social connection, the interaction was further interrogated to assess the direction of the effects.

Naltrexone (*vs* placebo) did not alter feelings of social connection to the cool object [*M placebo* = 0.117, s.d. = 0.932; *M naltrexone* = 0.154, s.d. = 1.037; *t*(77) = 0.168, *P* = 0.434]. Naltrexone, however, did move feelings of social connection to the warm object in the expected direction, but the reduction did not reach statistical significance [*M placebo* = 1.792, s.d. = 1.069; *M naltrexone* = 1.427, s.d. = 1.155; *t*(77) = 1.456, *P* = 0.075]. There were no associations between feelings of social connection and neural activity to the physical warmth task (*Ps* > 0.200).

The effects of naltrexone on ratings of thermal intensity were also evaluated. As expected, the warm stimuli were rated as significantly warmer than the cool stimuli [*t*(78) = 26.597, *P* < 0.001]. However, in a replication of our previous findings (Inagaki *et al.,* 2015), there was no effect of naltrexone on perceptions of thermal intensity for either the warm [*t*(77) = 1.030, *P* = 0.153] or cool objects [*t*(77) = 1.007, *P* = 0.159], suggesting naltrexone did not alter the more objective perceptions of warmth.

### Shared neural activity: social and physical warmth

**Fig. 4:**
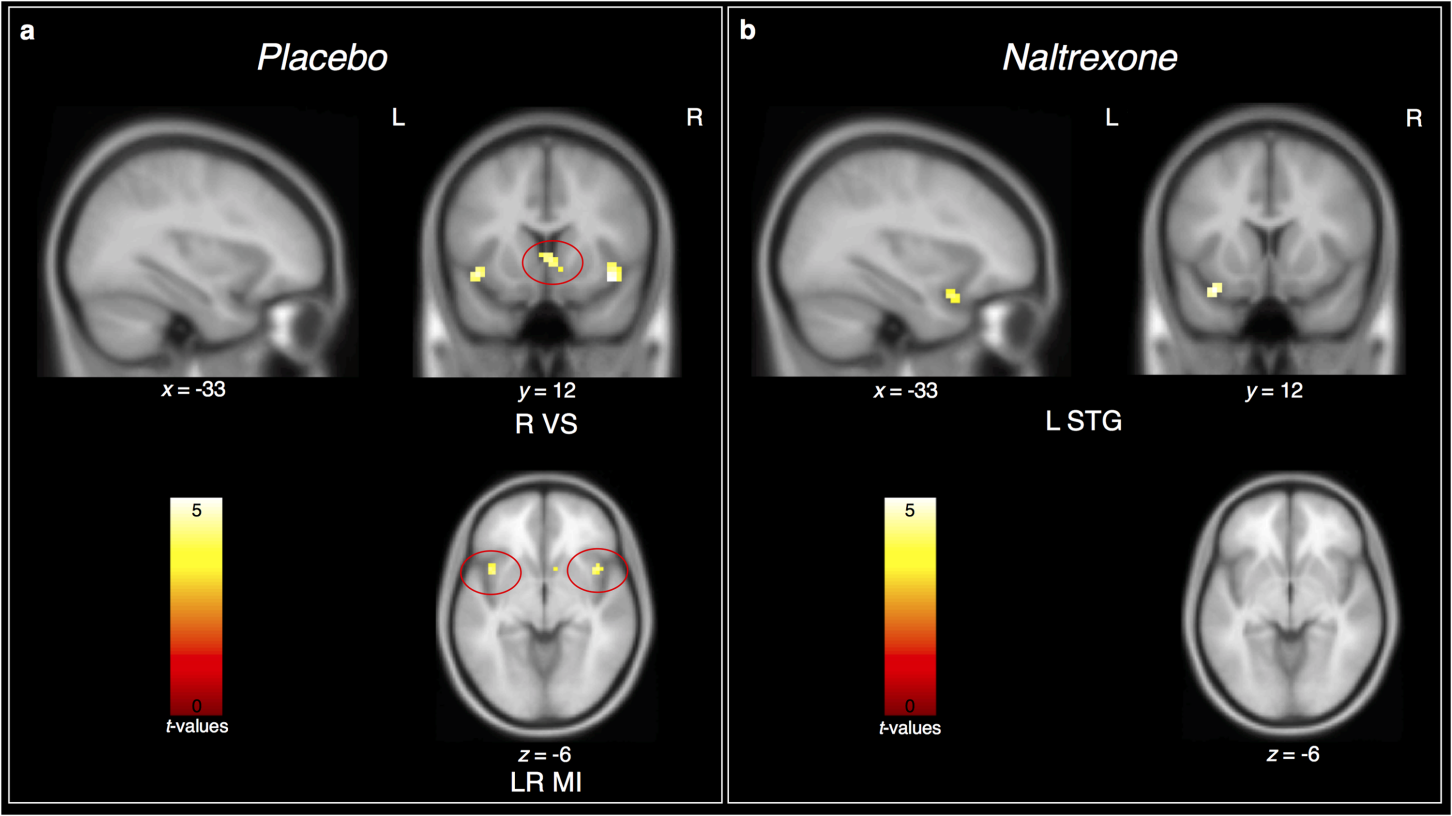
Results for conjunction between social warmth (messages from close others $>$ strangers) and physical warmth (warm $>$ neutral). In the placebo group (panel A), the only regions to show overlapping activity between social and physical warmth were the caudate, extending into the right VS, and left and right MI. In the naltrexone group (panel B), however, the only overlapping activity was in the left superior temporal gyrus. L = left, R = right, VS = ventral striatum, MI = middle insula, STG = superior temporal gyrus.

The hypothesized shared neural mechanisms between social and physical warmth were evaluated with conjunction analyses. In a replication of our previous findings, the conjunction analysis revealed shared neural activity in the left [*x* = −42, *y* = 12, *z* = −9, *t(*36*)* = 4.446, *k* = 24] and right MI [*x* = 42, *y* = 12, *z* = −12, *t(*36*)* = 4.855, *k* = 27] and caudate, extending into the right VS [*x* = 3, *y* = 18, *z* = 3, *t(*36*)* = 4.599, *k* = 29] to messages from close others (*vs* strangers) and the warm (*vs* neutral) object (Figure 4). In those who took naltrexone, however, there was no such overlap. Indeed, the only region to show overlapping activity between social and physical warmth was the left superior temporal gyrus [*x* = −33, *y* = 12, *z* = −21, *t(*39*)* = 4.235, *k* = 17].

## Discussion

How do we feel close and connected to our loved ones? The present study is the first to show that the opioid antagonist, naltrexone, reduces VS and MI activity to both social and physical warmth and erases the neural overlap between the two experiences. Together, these results advance two major theoretical perspectives on social bonding: (i) the brain opioid theory of social attachment and (ii) the notion that social and physical warmth share similar mechanisms. Each is elaborated on in turn.

### Brain opioid theory of social attachment

The brain opioid theory of social attachment suggests that blocking opioid activity would subsequently alter social connection (Panksepp *et al.,* 1980a; Panksepp, 1998). Consistent with some research findings on social behavior in animals, blocking opioids (*vs* placebo) in the current study reduced activity in the VS and MI to messages from participants’ own close others. In addition, naltrexone erased the positive correlation between feelings of social connection toward messages from close others and neural activity that was present in the placebo group. These results are the first to show that naltrexone causes a disconnect between subjective feelings of social connection and neural activity to reading messages that affirm one is loved, cared for, known and appreciated by their own loved ones.

Although the drug effect on self-reported feelings of social connection to the social warmth task was in the expected direction, results did not reach statistical significance. In everyday life, meaningful messages from close others, like the ones presented in the current study, are rare. Therefore, any feelings of social connection experienced after reading such messages may not be reduced (at a statistically significant level) by a single drug administration. Indeed, in our previous work with naltrexone and the same social warmth task, naltrexone did not have an effect on feelings of social connection during the first presentation of messages (Inagaki *et al.,* 2016a). Instead, the current results suggest that opioids may have a more nuanced effect on social connection experiences by not only altering neural responding but also disrupting the link between self-reported subjective experience and neural activity. Future work may wish to examine additional neurochemical contributors to self-reported feelings of social connection [e.g. oxytocin (Bartz, 2016), dopamine (Atzil *et al.,* 2017)] and their interactions with opioids as they contribute to subjective social experience. However, taken together, the results on the effects of naltrexone to the social warmth task add to a growing literature to suggest that the central actions of opioids alter experiences of social connection in humans (Machin and Dunbar, 2011; Nummenmaa *et al.,* 2015; Inagaki, 2018).

### Social and physical warmth

The present research contributes additional data in support of the notion that social warmth and the feelings of connection that can result from interacting with our closest others may act on the same pathways as physical warmth. An emerging literature shows that physical warmth can cause increases in feelings of social connection and that feelings of social connection can likewise increase feelings of warmth (e.g. Inagaki and Eisenberger, 2013). Responses to social and physical warmth in the current study further suggest a link in two ways. First, holding a warm (*vs* cool) object increased feelings of social connection and VS and MI activity. That is, simply holding a warm object was sufficient to temporarily increase feelings of social connection and VS and MI activity. Second, neural responding to social and physical warmth showed overlapping activity in the VS and MI, regions previously associated with close social bonds (Bartels and Zeki, 2004; Aron *et al.,* 2005; Acevedo *et al.,* 2012; Inagaki and Eisenberger, 2013; Atzil *et al.,* 2017).

Naltrexone, however, disrupted the neural patterns present in the placebo condition. First, naltrexone reduced VS and MI activity to physical warmth. Second, and perhaps more notable, naltrexone altered neural responding such that the overlap in responding to social and physical warmth that was present in the placebo group was eliminated. Together, these results suggest that another route by which opioids contribute to social bonding is via its effects on physical warmth.

### Implications for social bonding and relationship maintenance

Close social relationships that one maintains over time are the most critical relationships to health and well-being (e.g. Agerbo *et al.*, 2013). The current results have implications for understanding how opioids contribute to the maintenance of close social bonds. Specifically, results support the theory that opioids might affect thermoregulatory pathways [afferent central nervous system (CNS) pathways that bring the physiological condition of the body to the brain] and thus thermoregulation, the process by which the body maintains its temperature around a normotensive range, but not set point, or the level at which warm is considered warm (Adler *et al.,* 1988; Panksepp, 1998). In the current study, the thermoregulatory pathways tested include the VS and MI and the action of naltrexone on these brain regions. It is important to highlight that additional research that directly measures thermoregulation and its correlates (e.g. core body temperature, fever and shivering) as they contribute to social connection is needed. However, consistent with our theoretical perspective, naltrexone left perceptions of how warm the stimuli were rated (i.e. thermal intensity) unaltered but instead affected the more subjective experience of warmth. A possibility that could be tested with additional research is that the subjective experience of social bonding (feelings of social connection) is maintained similarly to how the thermoregulatory system maintains core temperature around a set point. In other words, feelings of social connection may fluctuate around a normotensive range, with the endogenous opioid system assisting in this maintenance. There is at least one study suggestive of a relationship between thermoregulation and the maintenance of affective experience. A single dose of whole-body heating (*vs* sham procedure) in individuals suffering from depression produces improvements in depressive symptoms both 1 week and 6 weeks after treatment, suggesting a long-term effect (Janssen *et al.,* 2016).

Also consistent with the notion that opioids contribute to the maintenance of close social bonds, naltrexone affected neural responses to messages from some of our participants’ own close others (e.g. parents, friends, romantic partners). However, naltrexone did not alter similar neural responses to messages from strangers. In a similar vein, naltrexone altered neural responses to warm stimuli, which may signal the proximity of close others but not cool stimuli (for the ROI results only). We have previously shown that the effects of naltrexone can be separated from general pleasant responding to both social warmth (Inagaki *et al.,* 2016a) and physical warmth (Inagaki *et al.,* 2015). The current results further clarify the contribution of opioids to social connection to suggest that opioids alter neural activity toward close others and related stimuli that might signal the presence of close others but not toward those one is not close to. These results not only underscore the similarities between social and physical warmth but also highlight an unrecognized and understudied side effect of opioid antagonism, namely altered neural responses to one’s own close others. If opioids affect VS and MI activity to one’s own close others after a single experience with these individuals (i.e. the social warmth task in the current study), it is possible opioids affect experiences that underlie bonding over time. However, as noted above, additional research that examines the long-term effects of weeks or months of naltrexone treatment are needed to directly assess the effects of naltrexone on relationships with close others over time.

Some limitations warrant discussion. The effect of naltrexone on self-reported feelings of social connection to social and physical warmth resulted in a pattern consistent with hypotheses but was not statistically significant. Indeed, the effect of naltrexone on feelings of social connection shows mixed results with some studies showing a statistically significant reduction (Inagaki *et al.,* 2015; Inagaki *et al.,* 2016a), some showing no effect (Tarr *et al.,* 2017) and some showing an effect only after considering moderators (Depue and Morrone-Strupinsky, 2005). Similarly, holding a warm *vs* a cool object did not increase VS and MI activity corrected at the whole brain in the placebo group. However, holding a warm object compared to a neutral, room temperature object increased VS and MI activity, in line with primary hypotheses and in a replication of our previous findings (Inagaki and Eisenberger, 2013). Additional research with larger samples and tighter control over the thermal stimulation may be needed to understand the effect of naltrexone on subjective social feelings toward close others and tease apart the brain’s response to warmer *vs* cooler temperatures.

In conclusion, this is the first study to show that naltrexone, an opioid antagonist, disrupts experiences of social connection by reducing VS and MI activity to social and physical warmth. Further, the effect of naltrexone was specific to close others and warmth, as naltrexone did not alter neural responses to strangers or cool stimuli. Within its limitations, the results contribute to an understanding of opioids and social bonding via two converging theories: the brain opioid theory of social attachment and a social-physical warmth overlap.

## Funding

The study was supported by a NARSAD Young Investigator Grant from the Brain & Behavior Foundation awarded to T.K.I., National Institute of Mental Health (NIMH) grants R01 MH 108509 and R01 MH076079 to C.A. and by the National Institute of Health (NIH) grant UL1TR001857. The funders were not involved in study design, collection of data, or manuscript preparation.

## Supplementary Material

supplementarymaterialsR2_nsz026
