# Supplementary material for: Naltrexone alters responses to social and physical warmth: implications for social bonding
Source: Soc Cogn Affect Neurosci. 2019 Apr 12;14(5):471–9. doi: 10.1093/scan/nsz026 (PMC6545530; doi:10.1093/scan/nsz026)
Supplement: supplementarymaterialsR2_nsz026 [file supplementarymaterialsr2_nsz026.docx]

Naltrexone alters responses to social and physical warmth:

implications for social bonding

Tristen K. Inagaki, Laura I. Hazlett, & Carmen Andreescu

Supplementary Material

Method

*Screening Procedures*

Interested individuals were telephone screened prior to an in-person visit. Inclusion criteria required participants to be in good health, between 18 and 35 years old, fluent in English, and right-handed (for the fMRI scan). Participants were excluded for major self-reported mental and physical illness (e.g., hepatic illness), depressive symptoms (>9 on the PHQ; Spitzer, Kroenke, & Williams, 1999), excessive alcohol use, medication aside from birth control, a positive urine pregnancy or drug test ((Opiates, Tetrahydracannabinol (THC), Cocaine, Amphetamines (AMP), Methamphetamines (mAMP)) both at the time of screening and before drug administration, a BMI greater than 35, and non-removable metal in the body or claustrophobia (for the fMRI scan). The study physician (CA) provided final approval of participants.

*Pre-session Message Collection*

Prior to the experimental session, participants nominated 8 close others. Participants nominated friends (63%; e.g., best friends, roommates), family members (29%; e.g., parents, siblings, grandparents), and romantic partners (8%). The experimenters then contacted the close others via email to help create the social warmth task. Emails explained that the experimenters were interested in exploring reactions to messages from friends, family members, and strangers and requested that the close other respond with 12 brief (1-2 sentences) messages addressed to the participant. Example messages were provided to guide the close others in their writing. Close others were asked not to tell the participant what the experimenters had emailed them to keep the participant unaware of the study goals. During a manipulation check, participants reported 100% compliance with this request. Average ratings of closeness (on a 1-not at all close to me, to 10-extremely close to me, scale) suggested the selected individuals were indeed close (*M* = 8.285, *SD* = .893). Once 5 close others had responded to the original email request, personalized social warmth tasks were made for each participant for presentation in the fMRI scanner.

*Experimental Session*

*Neuroimaging Measures*

In addition to the Social and Physical Warmth tasks, participants completed a modified version of a picture viewing task (Aron, Fisher, Mashek, Strong, & Brown, 2005) during which time they viewed images of people they know and people they do not know. In between blocks of images, participants completed mental serial subtraction. Results from this task are reported separately.

*Self-report Measures*

*Physical Symptoms*

Physical symptoms commonly experienced when taking naltrexone (Ray, Chin, & Miotto, 2010) were assessed before and after drug administration. Specifically, participants reported on headaches, dizziness/faintness, stomach discomfort, nausea, and tiredness/fatigue on a 0 (no symptoms) to 4 (very severe symptoms) scale. The highest reported value for any physical symptom, in either condition, was a 2. In addition, to assess the subjective experience of physical symptoms, participants were also asked, “overall, how distressing do you find these symptoms?” on a 1 (not distressing at all) to 7 (very distressing) scale.

*Whole Brain Analyses*

In addition to ROI analyses, neural activity across the whole brain was examined as a manipulation check, confirmation of the ROI analyses, and a replication attempt of our previous neuroimaging study using the same tasks (Inagaki & Eisenberger, 2013). Activity to the primary contrasts of interest from the social warmth task (messages from close others > messages from strangers) and physical warmth task (warm > neutral and warm > cool) were examined in the placebo group. Results were interrogated using a False Discovery Rate (FDR) of 0.05 with a cluster size threshold of 16 voxels to correct for multiple comparisons. Coordinates are reported in Montreal Neurological Institute (MNI) format.

Results

*Study Drug Blind and Physical Symptoms*

Participants were not able to guess which drug they had been given above chance levels (50%) in either the placebo (*X^2^*(1) = 2.489, *p* = .115) or naltrexone condition (*X^2^*(1) = 1.766, *p* = .184). In addition, physical symptoms were greater for those in the naltrexone condition (*M* = .425, *SD* = .357) than placebo condition (*M* = .210, *SD* = .293, *t*(78) = 2.942, *p* = .001) post-drug administration. The same pattern emerged for distress of the symptoms such that distress from the symptoms was greater in the naltrexone (vs. placebo) condition (*t*(78) = 2.014, *p* = .024). Feelings of social connection to the social and physical warmth task remain unchanged when controlling for physical symptoms or distress of the symptoms.

*Neural Activity to Social Warmth Task-whole brain corrected*

Replicating our previous results with the social warmth task and as confirmation of the ROI analyses, there was extensive activity in the ventral striatum (VS) and middle-insula (MI). In addition, there was greater activity in regions of the social cognition network (temporal pole, dorsomedial prefrontal cortex; Mitchell, Macrae, & Banaji, 2004), regions implicated in affiliative responding (dorsal, mid, and posterior cingulate cortex, ventral tegmental area, septohypothalamic regions; Moll et al., 2012), and the cerebellum to messages from close others as compared to messages from strangers. For a full list of activations, see Supplementary Table 1.

*Neural Activity to Physical Warmth Task-whole brain corrected*

In a replication of our previous results and previous studies on the neural response to warm, cutaneous stimuli (Becerra et al., 1999; Davis et al., 1998; Olausson et al., 2005; Rolls et al., 2008), holding a warm (vs. neutral) object lead to increased activity in the left and right MI and the caudate (extending into the VS; see supplementary Table 3 for a full list of activations). Thus, warmth increased activity in the VS and MI, consistent with the notion that warmth might contribute to social connection. No regions showed greater activity to the warm than cool pack at the current threshold.

Discussion

Results from analyses corrected at the whole brain align with the ROI results reported in the parent manuscript to confirm that the social and physical warmth tasks lead to increased activity in the VS and MI in the placebo condition. Of note, neural activity to reading messages from close others (vs. strangers) resulted in a large activation with peaks in the cerebellum, regions of the social cognition network, and regions previously implicated in affiliative responding. Though traditionally associated with motor movement, the cerebellum may, in the current context where participants read messages from close others, reflect reading and interpreting the messages (Buckner, 2013). Indeed, in a meta-analysis of the role of the cerebellum in social cognition, cerebellum activity is high when the level of abstraction is also high (Van Overwalle, Baetens, Marien, & Vandekerckhove, 2014). An intriguing additional possibility, based on recent findings in mice, is that cerebellum activity in response to the messages from close others reflects social reward (Carta, Chen, Schott, Dorizan, & Khodakhah, 2019).

References

Becerra, L. R., Breiter, H. C., Stojanovic, M., Fishman, S., Edwards, A., Comite, A. R., . . . Borsook, D. (1999). Human brain activation under controlled thermal stimulation and habituation to noxious heat: An fMRI study. *Magnetic Resonance in Medicine, 41*, 1044–1057.

Buckner, RL (2013). The cerebellum and cognitive function: 24 years of insight from anatomy and neuroimaging. *Neuron, 80*, 807-15.

Carta, I., Chen, C. H., Schott, A. L., Dorizan, S., & Khodakhah, K. (2019). Cerebellar modulation of the reward circuitry and social behavior. *Science, 363*.

Davis, K. D., Kwan, C. L., Crawley, A. P., & Mikulis, D. J. (1998). Functional MRI study of thalamic and cortical activations evoked by cutaneous heat, cold, and tactile stimuli. *Journal of Neurophysiology, 80*, 1533–1546.

Inagaki, T. K., & Eisenberger, N. I. (2013). Shared neural mechanisms underlying social warmth and physical warmth. *Psychological Science*, *24*, 2272-2280.

Mitchell, J. P., Macrae, C. N., & Banaji, M. R. (2004). Encoding-specific effects of social cognition on the neural correlates of subsequent memory. *Journal of Neuroscience*, *24*, 4912-4917.

Moll, J., Bado, P., de Oliviera-Souza, R., Bramati, I. E., Lima, D. O., Paiva, F. F., . . . Zahn, R. (2012). A neural signa- ture of affiliative emotion in the human septohypothalamic area. *Journal of Neuroscience,* *32*, 12499–12505.

Olausson, H., Charron, J., Marchand, S., Villemure, C., Strigo, I. A., & Bushnell, M. C. (2005). Feelings of warmth correlate with neural activity in right anterior insular cortex. *Neuroscience Letters, 389*, 1–5.

Ray, L. A., Chin, P. F., & Miotto, K. (2010). Naltrexone for the treatment of alcoholism: clinical findings, mechanisms of action, and pharmacogenetics. *CNS Neurol Disord Drug Targets, 9,* 13-22.

Rolls, E. T., Grabenhorst, F., & Parris, B. A. (2008). Warm pleasant feelings in the brain. *Neuroimage*, *41*, 1504-1513.

Spitzer, R. L., Kroenke, K., & Williams, J. B. (1999). Patient Health Questionnaire Primary Care Study Group. Validation and utility of a self-report version of PRIME-MD: the PHQ primary care study. *JAMA, 282,* 1737-44.

Van Overwalle, F., Baetens, K., Mariën, P., & Vandekerckhove, M. (2014). Social cognition and the cerebellum: a meta-analysis of over 350 fMRI studies. *Neuroimage*, *86*, 554-572.

Fig. 1

**CONSORT Flow Diagram**

## Enrollment

Randomized (n=82)

Assessed for eligibility (n=150)

Excluded (n=68)

♦  Not meeting inclusion criteria (n=13)

♦  Declined to participate (n=14)

♦  Other reasons

- Lack of compliance (*n*=41)

## Allocation

Allocated to naltrexone condition (n=42)

♦ Received naltrexone (n=42)

♦ Did not receive naltrexone (n=0)

Allocated to placebo condition (n=40)

♦ Received placebo (n=40)

♦ Did not receive placebo (n=0)

Analysed (n=40)

♦ Excluded from self-report analysis (n=2)

- Did not follow instructions for ratings to social warmth task (n=1)
- Misplaced ratings for physical warmth task (n=1)

♦ Excluded from fMRI analysis (n=3)

- Removed from the scanner in between the structural scan and the functional scans resulting in poor registration during preprocessing (n=1)
- Technical error with task presentation; (*n*=1)
- Signal dropout in the primary region of interest (n=1)

## Analysis

Analysed (n=40)
♦ Excluded from all analyses (n=2)

- Could not complete fMRI scan due to severe symptoms (n=1)
- Could not complete fMRI scan due to head size (n=1)

♦ Excluded from self-report analysis (n=1)

- Misplaced ratings

| Table 1. Brain regions more active when reading messages from close others compared to reading messages from strangers | | | | | | | |
| --- | --- | --- | --- | --- | --- | --- | --- |
|  |  |  | MNI  coordinates | | |  |  |
| Anatomical Region | Hemisphere | BA | x | y | z | *t* | *k* |
| Cerebellum |  |  | 24 | -78 | 36 | 11.49 | 17707 |
| Ventral Striatum | R |  | 15 | 0 | -6 | 8.50 |  |
|  | L |  | -12 | 0 | -9 | 8.16 |  |
| Middle insula | L |  | -39 | 9 | -12 | 5.61 |  |
|  | R |  | 45 | 9 | -12 | 3.60 |  |
| Ventral Tegmental Area | R |  | 9 | -12 | -21 | 3.84 |  |
|  | L |  | -9 | -15 | -18 | 4.79 |  |
| Septal Area |  |  | 0 | 3 | -3 | 5.99 |  |
| Hypothalamus |  |  | -3 | -9 | 0 | 4.53 |  |
| Hippocampus | L |  | -30 | -9 | -15 | 5.71 |  |
|  | R |  | 30 | -9 | -12 | 6.18 |  |
| Dorsal anterior cingulate cortex | L | 32 | -3 | 33 | 30 | 6.74 |  |
|  | R | 32 | 3 | 33 | 30 | 6.30 |  |
| Mid-cingulate cortex |  | 24 | 0 | -15 | 36 | 9.29 |  |
| Posterior cingulate cortex | L | 31 | -6 | -51 | 30 | 9.25 |  |
| Dorsomedial prefrontal cortex | R | 10 | 6 | 57 | 21 | 8.16 |  |
| Medial prefrontal cortex | L | 10 | -3 | 60 | 18 | 10.55 |  |
| Temporal Pole | L | 38 | -51 | 12 | -21 | 4.33 |  |
|  | R | 38 | 51 | 12 | -21 | 5.44 |  |
| Inferior frontal gyrus | L | 47 | -36 | 21 | -15 | 10.63 |  |

Note: Activations significant at FDR .05, 16 voxels. L/R=left and right hemispheres; BA=Brodmann’s Area; x, y, and z=Montreal Neurological Institute (MNI) coordinates; *t*=t statistic value at peak coordinates; *k*=cluster voxel extent; Activations that do not include a *k*-value extend from the larger cluster listed above those activations.

| Table 2. Descriptive Statistics from the interaction between drug and social target for feelings of social connection to the social warmth task | | |
| --- | --- | --- |
|  | Placebo | Naltrexone |
| Messages from Close Others | 4.766 (.895) | 4.576 (.848) |
| Messages from Strangers | 1.906 (.659) | 2.026 (.781) |

| Table 3. Brain regions more active when holding a warm compared to holding a room temperature object | | | | | | | |
| --- | --- | --- | --- | --- | --- | --- | --- |
|  |  |  | MNI coordinates | | |  |  |
| Anatomical Region | Hemisphere | BA | x | y | z | *t* | *k* |
| Caudate extending into Ventral Striatum | L/R |  | 3 | 18 | 3 | 4.60 | 45 |
| Posterior Insula | R |  | 45 | -15 | 18 | 5.55 | 403 |
| Anterior/Mid-Insula | R |  | 36 | 9 | 12 | 4.33 |  |
| Mid-Insula | R |  | 42 | 12 | -12 | 4.86 |  |
|  | L |  | -39 | 9 | 9 | 4.57 | 122 |
| Primary motor cortex | L | 4 | -60 | 6 | 9 | 3.91 | 17 |

Note: Activations significant at FDR .05, 16 voxels. L/R=left and right hemispheres; BA=Brodmann’s Area; x, y, and z=Montreal Neurological Institute (MNI) coordinates; *t*=t statistic value at peak coordinates; *k*=cluster voxel extent; Activations that do not include a *k*-value extend from the larger cluster listed above those activations.
